# Lycopene: A Potent Antioxidant with Multiple Health Benefits

**DOI:** 10.1155/2024/6252426

**Published:** 2024-06-08

**Authors:** Mercy Omoye Shafe, Nontobeko Myllet Gumede, Trevor Tapiwa Nyakudya, Eliton Chivandi

**Affiliations:** ^1^School of Physiology, Faculty of Health Sciences, University of the Witwatersrand, 7 York Road, Parktown, Johannesburg 2193, South Africa; ^2^Department of Human Physiology, Faculty of Basic Medical Sciences, College of Medicine and Allied Health Sciences, Bingham University, P.M.B. 005, New Karu, Nasarawa 961002, Nigeria; ^3^Department of Physiology, School of Medicine, Faculty of Health Sciences, University of Pretoria, Private Bag X323, Gezina, Pretoria 0031, South Africa

## Abstract

Lycopene is a naturally occurring carotenoid predominantly found in tomatoes and tomato-based products. Like other phytochemicals, it exhibits health beneficial biological activities that can be exploited when it is used as a dietary supplement. *In vitro* and *in vivo*, lycopene has been demonstrated to mitigate oxidative stress-induced metabolic dysfunctions and diseases including inflammation, obesity, and diabetes mellitus. Lycopene has been shown to alleviate metabolic diseases that affect the bone, eye, kidney, liver, lungs, heart, and nervous system. This review presents the state of the art regarding lycopene's health benefits and its potential applications in health system delivery. Furthermore, lycopene's protective effects against toxins, safety in its use, and possible toxicity are explored.

## 1. Introduction

The use of medicinal plants has deep historical roots, ingrained in the traditional healing practices of diverse cultures worldwide [1]. Throughout centuries, indigenous communities and ancient civilizations have harnessed the therapeutic properties of plants, passing down invaluable knowledge through generations [2]. Ethnomedicine, a field dedicated to study traditional medicinal practices, has played a crucial role in documenting this wealth of wisdom. The effectiveness of ethnomedicinal plants in disease management is attributed to their constituent bioactive phytochemicals, such as carotenoids, which are known to have multiple health benefits [3]. Lycopene, a fat-soluble carotenoid, is one of the most abundant and important carotenoids [4]. It has potent antioxidant activity [5]. This carotenoid, a bioactive organic pigment, is found in pink grapefruit, papaya, guava, apricot, watermelon, and vegetables but is highly concentrated in tomatoes and tomato-derived products [6]. It has been reported to be one of the strongest antioxidants among carotenoids [7]. As one of the most potent antioxidants, its capacity to neutralise singlet oxygen is double that of *β*-carotene, ten times greater than that of *α*-tocopherol, and one hundred and twenty-five times more effective than glutathione [5]. Lycopene, isolated from *Lycopersicum esculentum* (tomato) in 1903, was named after the fruit from which it was isolated [8]. More than 85% of the lycopene in the diet is derived from tomatoes and tomato-based products [8]. In addition to fruits and vegetables, lycopene is also found in some food ingredients, as shown in Table 1 [9, 10]. While overall tomatoes are a good source of lycopene, research has demonstrated that different tomato and other fruit varieties have different lycopene content [7]. In addition to varietal differences, the microenvironment in which the tomato and or other lycopene-containing fruit are grown, for example, temperature, humidity, edaphic conditions, and fruit maturity status at harvest also influence lycopene content [11]. Where the soil microbiome has favourable microbes, a 36% increase in lycopene has been reported [11].

Several studies have investigated the potential of lycopene to mitigate risk factors for obesity, type 2 diabetes mellitus, and cardiovascular diseases, conditions characterised by dyslipidaemia, oxidative stress, and inflammation [12]. These studies have shown that lycopene improved outcomes of these metabolic diseases [13]. Lycopene, known for its antioxidant properties, has been found to reduce oxidative stress, a significant contributor to the development of metabolic diseases [14]. In addition, it has been shown to mitigate inflammation and dyslipidaemia, thereby reducing the risk of cardiovascular diseases and insulin resistance [15, 16]. Research suggests that regular consumption of lycopene as a dietary supplement can potentially remediate insensitivity to insulin, hypertension, and obesity-related metabolic complications [17, 18].

## 2. Lycopene: Biochemistry and Physical Properties

**Figure 1:**
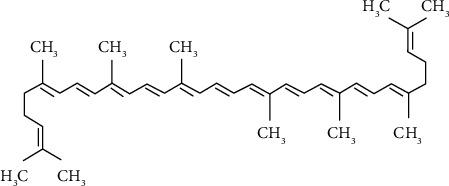
Molecular structure of lycopene.

In nature, over 750 carotenoids have been identified [19]. About 40 to 50 are found in the human diet, and lycopene is the sixth most common carotene in food products [20, 21]. Two main categories of carotenoids exist: hydrocarbon carotenoids and xanthophylls. Hydrocarbon carotenoids such as *α-*, *β*-, and *γ*-carotene lycopene are made up of hydrogen and carbon, while xanthophylls, for example, lutein, *β*-cryptoxanthin, and zeaxanthin, contain oxygen along with carbon and hydrogen [4, 22]. Lycopene, as an aliphatic straight-chain hydrocarbon, contains two unconjugated double bonds and 11 conjugated bonds [23]. Its conjugated double bonds are subject to isomerization through heat, light, and chemical reactions [20]. Lycopene is found in *trans*- and *cis*-isomers, but the *cis*-isomers are better absorbed and have greater bioavailability than *trans*-lycopene [24, 25]. All-*trans*, 5-*cis*, 9-*cis,* 13-*cis*, and 15-*cis* are the most common forms of lycopene isomers, and the 5-*cis* isomer is the most stable isomer [26, 27]. The molecular structure and physical properties of lycopene are shown in Figure 1 [28] and Table 2, respectively [8, 29].

## 3. Lycopene: Absorption, Transportation, and Distribution

Following ingestion, lycopene released from the food matrix combines with micelles-containing bile salts, cholesterol, and fatty acids [30] and is then absorbed. Due to its hydrophobicity, the dissolution of lycopene within micelles in the small intestines facilitates its absorption [5] through the passive diffusion of lipids across the unstirred water layer in the enterocytes [31]. Inside the absorptive enterocyte, lycopene, together with free fatty acids, monoglycerides, and fat-soluble vitamins, is packaged into chylomicrons and released into the lymphatic system for transportation into the bloodstream and liver [23]. A fibre-rich diet has been proven to decrease the absorption of lycopene. Such fibrous diets also mediate the absorption of lycopene, resulting in over 40% reduction in plasma lycopene [32]. Several factors, among these, alcohol, smocking, gender, age, hormonal status, and other dietary elements, affect the absorption of lycopene [32]. As healthy individuals grow older, the bioavailability of lycopene tends to decrease, possibly due to age-related structural changes in the gastrointestinal tract that result in reduced absorptive efficiency [33]. Humans absorb about 10% to 30% of dietary lycopene; the rest is excreted through faeces [8, 33]. The lycopene in heated and processed tomato products is better absorbed compared to that from fresh, unprocessed tomatoes [20]. Thermal exposure during cooking and processing of lycopene-containing foods breaks the food matrix and converts the natural (all-trans) lycopene structure to its *cis* geometric isomer, which is 2.5 times better absorbed from the gastrointestinal tract [34, 35]. Following its absorption from the small intestines, lycopene is distributed to the various body tissues [33]. The assimilation of lycopene by the tissues from lipoproteins is mediated by certain membrane receptors known as scavenger receptor class B type 1 (SR-B1) and cluster of differentiation 36 (CD36) [4]. In humans, the concentration of lycopene in the testes is ten times greater than that found in other tissues [8]. This high concentration in the testes is followed by its concentration in the adrenal gland, liver, prostate, breast, pancreas, skin, colon, ovary, lung, stomach, kidney, adipose tissue, and cervix [8]. However, *cis*-lycopene is mainly distributed in the liver and adipose tissue [24].  Table 3 illustrates the concentration of lycopene in various human tissues [36, 37]. Lycopene, the primary carotenoid found in human plasma, exhibits a half-life of approximately 2 to 3 days. Its concentration in plasma and tissues ranges between 0.2–21.4 nmol/g and 0.15–21.36 nmol/g, respectively [8, 36]. In their study, Zaripheh et al. [38] reported that in rats, lycopene was most concentrated in the liver, adipose tissue, adrenal tissue, and spleen.

## 4. Lycopene Autoxidation

Known to be thermolabile, lycopene undergoes autoxidation when exposed to both light and oxygen [23]. The heat-, light-, and oxygen-induced lycopene degradation gives rise to acetone, methyl-heptenone, laevulinic aldehyde, and glycoxal, a colourless compound that produces a grass-like smell [23]. In addition to the attractive colour of the final lycopene degradation products, their biodegradation also affects their flavour and nutritive value [39].

## 5. Biological Activities of Lycopene

The meta-analyses and clinical trials of lycopene in human studies are shown in Table 4.

### 5.1. Antiobesity Effects

Obesity results from an excessive buildup of body fat. It has a detrimental effect on a person's metabolic health and overall well-being [66]. The development of obesity is influenced by a variety of factors with complicated origins that involve psychological, environmental, socioeconomic status, and biological components [67–69]. The risk of cardiovascular diseases, cancer, depression, dyslipidaemia, type 2 diabetes mellitus, nonalcoholic fatty liver diseases (NAFLD), and hypertension is heightened in obese individuals [70–73]. Obesity elevates the prevalence of oxidative stress by disrupting the balance between oxidants and antioxidant activity [74], which leads to the presence of “unpaired mitochondria” (individual mitochondria within a cell that have not fused or aligned with others to form interconnected networks) and an upsurge in reactive oxygen species [75]. Consequently, the normal functioning of the adipose tissue is disrupted, resulting in an increased production of adipocytokines and a reduction in adiponectin levels, which contribute to the occurrence of metabolic syndrome [76, 77]. Numerous studies have reported on the health beneficial antioxidant activity of lycopene. In male Wistar rats exposed to a high-fat diet for 12 weeks, supplementation with lycopene at 25 mg/kg body weight for a period of 4 weeks was shown to reduce plasma interleukin 6 (IL-6), tumour necrosis factor alpha (TNF-*α*), leptin, very low-density lipoprotein (VLDL), low-density lipoprotein (LDL), and total cholesterol (TC), but it elevated plasma high-density lipoprotein (HDL) levels [78]. The supplemental lycopene also reduced malondialdehyde (MDA) concentration but increased hepatic superoxide dismutase (SOD) and catalase (CAT) activities in the liver tissue, demonstrating that it (lycopene) potentially is a potent antioxidant that decreases hepatic oxidative stress by increasing systemic antioxidant and enzyme activities [78]. Pre- and/or postweaning supplementing Sprague–Dawley rat pups whose dams were fed a high-fat diet with lycopene at 1% improved the offspring's brown adipose tissue (BAT) development, reduced accumulation of white adipose tissue (WAT), and enhanced serum antioxidant capacity and blood glucose homeostasis [79]. In mice fed a high-fat diet, lycopene was shown to improve glucose and lipid metabolism and decrease body weight gain by stimulating WAT browning and activating BAT through modulation of peroxisome proliferator-activated receptor gamma (PPARG) [24]. In another study, where lycopene was administered at 25 and 50 mg/kg body weight for 3 months to male Wistar rats, results showed increased HDL, improved antioxidant, and oxidant biomarkers, decreased triglycerides (TG), LDL, apolipoprotein-B (Apo-B), and *β*-hydroxybutyrate, but boosted hepatic PPARG levels [80]. Furthermore, tomato oleoresin, which contains 10 mg/kg body weight of lycopene, when orally administered to male Wistar rats for 6 weeks, mediated a significant increase in the expression of messenger RNA (mRNA) of adiponectin, forkhead box 01 (Fox01), fatty acid translocase/cluster of differentiation 36 (FAT/CD36), and sirtuin 1 (SIRTI), but downregulated PPARG expression in the adipose tissue of obese rats [81].

### 5.2. Antioxidant Effects

Oxidative stress is recognised as a significant contributing factor to an increased risk of cancer, the onset and progression of various metabolic and chronic disorders [82]. The concept of oxygen radicals has been established for the past 50 years; however, its role in the advancement of diseases was discovered in the past two decades [83]. In several biological processes that are vital for life, free radicals play an important role, such as the destruction of intracellular bacteria by phagocytes such as macrophages and granulocytes [82]. Excessive production of reactive oxygen species (ROS) causes protein, deoxyribonucleic acid (DNA), and lipid damage [84]. Damage to these cellular molecules leads to tissue injury and interruption in vital cellular processes [85]. Consuming diets rich in antioxidants or supplementing with bioactive molecules such as vitamins, tannins, and carotenoids may offer protection against oxidative damage [86]. Carotenoids such as lycopene are potent antioxidants that inhibit or hinder the advancement of diverse disorders triggered by ROS [5]. Carotenoid antioxidant activity has been investigated in multilamellar liposomes by measuring the inhibition of the formation of thiobarbituric acid-reactive substances. Lycopene was shown to be the most potent antioxidant in the sequence: lycopene, ϒ-tocopherol, astaxanthin, canthaxanthin, *α*-carotene, *β*-carotene, bixin, zeaxanthin, lutein, *α*-tocopherol, glutathione, cryptoxanthin, crocin, and lipoic acid [8, 87]. Lycopene attenuates ROS effects through radical addition or adduct formation, electron transfer to the radical, and allylic hydrogen abstraction [6], and radical addition and allylic hydrogen abstraction contribute to its antioxidant effects [88]. Lycopene has been reported to enhance the status of enzymatic (catalase, superoxide dismutase, and peroxidase) and nonenzymatic antioxidants such as vitamins C and E from their radicals by increasing the cellular antioxidant defence system [33]. In addition, lycopene acts as an antioxidant in systems that produce singlet oxygen but behaves as a pro-oxidant in systems that create peroxide [89]. In low doses, it acts as an antioxidant, but at high doses, it acts as a pro-oxidant [90]. Factors such as lycopene concentration, tissue oxygen tension, and interaction with other antioxidants have been reported to influence the pro-oxidant potency of lycopene [6]. In situation where there is an imbalance between antioxidant defences and ROS production, such as during inflammation or exposure to environmental toxins [91], lycopene may switch from its antioxidant role to a pro-oxidant role [89]. Under these conditions, lycopene radicals may contribute to oxidative stress by reacting with cellular components and promoting further ROS generation [92]. Studies have suggested that under conditions of low oxygen levels, its antioxidant properties predominate [93, 94].

### 5.3. Hypocholesterolaemic Effects

An imbalance in the level of cholesterol in the body results in a lipid disorder known as hypercholesterolemia, a notable risk factor for atherosclerosis and related conditions such as coronary and cerebrovascular diseases [95, 96]. Several animal and human trials have investigated the association between lycopene and cholesterol. Male broiler chickens fed a standard grower diet supplemented with lycopene at 100 mg/kg body weight for 3 weeks had significantly reduced serum total cholesterol, triglyceride, very low-density lipoprotein, and increased high-density lipoprotein content compared to counterparts fed the control diet [97]. In apolipoprotein E knockout mice fed a high-fat diet and lycopene supplementation at 60 mg/kg body weight daily for 14 weeks, the administered lycopene significantly decreased both total cholesterol and triglycerides, beginning from the sixth week to the end of the experiment [98]. Similarly, male Wistar rats given a high-fat diet and 50 mg/kg body weight of lycopene daily for 3 months had significant reductions in plasma total cholesterol, triglycerides, and low-density lipoprotein levels but increased high-density lipoprotein cholesterol compared to the group given a high-cholesterol diet [99]. The reported cholesterol-lowering effects of lycopene are attributed to reduce cholesterol synthesis through the inhibition of the expression and activity of 3-hydroxy-3-methylglutaryl coenzyme A (HMG-CoA) reductase and the modulation of LDL receptor activity [100]. The findings obtained from human studies have been inconsistent. In a systematic review and meta-analysis of 12 and 11 trial arms consisting of 781 and 854 participants, respectively, supplementation of lycopene significantly increased HDL-cholesterol levels when compared to the control group; however, no significant difference was observed in the triglyceride levels [101]. The conflicting findings observed from human studies could be attributed to the differences in the study design, characteristics of the populations under investigation, and the source and dose of lycopene utilised [16, 102].

### 5.4. Hepatoprotection

In a healthy human adult, the liver weighs approximately 1.5 kg and is the largest gland and visceral organ [103]. It plays a vital role in metabolic processes such as bile production, digestion, detoxification of xenobiotics, metabolism of lipids, proteins, carbohydrates, immune regulation, and storage of vitamins [104, 105]. Among the major causes of global mortality is liver disease [106]. Liver diseases may be caused by several factors, viral infections, ischemia, alcohol-induced damage, autoimmune diseases, and genetic defects such as alpha-1 antitrypsin deficiency, hereditary hemochromatosis, citrin deficiency, hereditary fructose intolerance, cystic fibrosis, cholesteryl ester storage disease, type IV glycogen storage disease, and Wilson disease [107–109]. Nonalcoholic fatty liver disease (NAFLD) is the most prevalent liver disease [110]. Globally, the prevalence of NAFLD is about 25%, in Africa, it is 13% while in Europe, the rate is 23% and the highest at 32% in the Middle East [111]. This disease is characterised by the accumulation of macrovesicular steatosis in ≥5% of hepatocytes without secondary causes such as alcohol intake, drugs, or liver diseases [111, 112]. Patients with type 2 diabetes, dyslipidaemia, and obesity are at increased risk of developing NAFLD [113]. Recent studies have shown that consumption of carotenoids such as lycopene can remarkably reduce the chances of developing liver diseases such as NAFLD [90]. In their study, Li et al. [114], using beta-carotene-15,15′-oxygenase and beta-carotene-9′,10′-oxygenase double knockout mice, the oral administration of lycopene at 2.3 mg/g for 24 weeks resulted in significantly decreased severity of hepatic steatosis and triglyceride levels but significantly increased sirtuin 1 and fatty acid oxidation compared to control counterparts fed a high-fat diet. Furthermore, lycopene mediated a decrease in inflammation. In a tramadol-induced hepatotoxicity rat model, supplemental lycopene at 15 mg/kg body weight for 15 days mitigated the hepatotoxicity by increasing antioxidant activity, reducing fatty acid breakdown and necrosis, lipid peroxidation, inhibiting DNA fragmentation, and apoptosis [115]. Lycopene administered at 5, 10, and 20 mg/kg body weight for 6 weeks in a rat model of NAFLD was shown to mediate hepatoprotective effects, as seen with reduced activities of aspartate transaminase and alanine transaminase and concomitant reductions in malondialdehyde, free fatty acids, and LDL-cholesterol concentrations [116]. These findings were associated with elevated hepatic superoxide dismutase and glutathione concentrations, but with reduced cytochrome P450 2E1 and tumour necrosis factor-alpha expression and decreased hepatic fat [116]. The abovementioned experimental studies provide a clear insight that the administration of lycopene not only inhibits ROS but also improves the activity of antioxidant enzymes, thereby providing beneficial effects against NAFLD.

### 5.5. Renoprotection

Chronic kidney diseases (CKD) have become a global public health issue, affecting more than 200 million people worldwide [117]. Chronic kidney disease is a common term used to describe different disorders that permanently affect the structure and function of the kidneys for over a period of 3 months [118]. This can be diagnosed when the abnormalities in the kidney or glomerular filtration rate are lower than 60 ml/min/1.73 m^2^ and albuminuria is characterised by an albumin to creatinine ratio above 30 mg/g [119]. Patients with CKD are more prone to develop end-stage renal disease, a condition that requires expensive management by either dialysis or kidney transplantation [76]. Patients suffering from CKD commonly display a high incidence of arrhythmias, venous thromboembolism, heart failure, and ischemic heart disease, which significantly increases mortality [120, 121]. The increase in the prevalence of cardiovascular disease (CVD) in CKD patients is associated with oxidative stress, chronic inflammation, and vascular endothelial dysfunction [122]. These three factors create an intricate cycle, resulting in pathological variations and playing a crucial role in the initiation and progression of CVD in CKD patients [123, 124]. Among these factors, oxidative stress is a key mediator in the intricate pathways linked to the progression of CKD [124]. As a result, the utilisation of antioxidant therapy is one of the significant approaches to avert and mitigate the advancement of CKD [56]. Lycopene is a potent antioxidant and an efficient free radical scavenger that has been investigated and shown to protect the kidney against chemically induced damage [125, 126]. In female Wistar rats fed a high-fat diet, the supplementation of 200 ml of lycopene extract twice a week for 8 weeks significantly reduced plasma creatinine, urea, serum angiotensin-converting enzymes, renal tissue malondialdehyde, and C-reactive protein levels but increased total protein and tissue antioxidant enzyme levels [127]. Tabrez et al. [128] observed that lycopene protected against the advancement of diabetic nephropathy and improved renal function by inhibiting the advanced glycation product and its receptors' (AGE-RAGE) pathway. Lycopene has shown to inhibit LDL-cholesterol peroxidation, which can damage the kidneys [56]. Furthermore, supplemental lycopene has shown to decrease MDA, RAGE, and TNF-*α* levels in the kidneys of male Wistar rats fed a high-fat diet for 6 weeks [129], and similarly, lycopene orally administered at 25 and 50 mg/kg body weight daily for 3 months protected the kidneys of male Wistar rats fed a high-fat diet by inhibiting the expression of nuclear factor kappa-B, interleukin 1 beta, tumour necrosis factor alpha, decreasing oxidative stress, increasing nuclear factor erythroid 2-related factor 2, and stimulating B-cell lymphoma 2, hence shielding the kidney tissue against damages [66].

### 5.6. Osteoprotection

Oxidative stress caused by reactive oxygen species influences the activity of both osteoclasts and osteoblasts [130]. This is thought to impact the pathogenesis of skeletal system disorders, including osteoporosis, the most common skeletal metabolic disease [131]. Osteoporosis often develops in older adults and is characterised by an alteration of the bone microarchitecture, typified by a decline in bone mineral density, which contributes to an elevated risk of fractures [132]. Such bone fractures notably occur at the distal forearm, vertebral column, and proximal femur [133]. Complications associated with osteoporosis, particularly hip fractures, result in a mortality rate that is 4 times higher in the global adult population [132]. Despite its preponderance in the elderly, osteoporosis has shown to impact individuals of various age groups, but postmenopausal women are at high risk [134, 135] due to a decrease in estrogen production which results in increased oxidative stress and osteoclast-induced bone resorption [136]. Studies have shown that children born to parents with a history of osteoporosis and fractures are more prone to the development of osteoporosis [137]. In addition to genetic predisposition, poor nutrition, excessive alcohol consumption, smocking, caffeine intake, and medication side effects, for example, glucocorticoids, can cause the development of osteoporosis [138–141]. Lycopene has shown to have an advantageous effect on the skeletal health [142]. It has shown to play a vital role in protecting postmenopausal women from experiencing bone loss by upregulating alkaline phosphatase, type 1A collagen, runt-related transcription factor 2, triggering the activation of the wingless-related integration site/beta-catenin and extracellular signal-regulated kinase 1/2 pathways, and downregulating receptor activator of nuclear factor kappa-B ligand [143]. In mice fed a high-fat diet, supplemental lycopene at 15 mg/kg body weight for 10 weeks increased serum levels of total antioxidant capacity (T-AOC), SOD, and reduced the levels of MDA and AGEs, RAGE, and NF-kB expressions in the tibias and femurs [144]. In male albino rats, orally administered lycopene at 30 mg/kg body weight once daily over an 8-week period mitigated glucocorticoid-induced osteoporosis [145], and in diabetic male rats, lycopene suppressed bone resorption, enhanced osteopreotegerin and RANKL expression ratios by preventing oxidative damage and reducing inflammation [146]. These research findings demonstrate that lycopene has osteoprotective properties.

### 5.7. Anti-Inflammatory Effects

Inflammation is an immune response mechanism that is triggered when exposed to various harmful stimuli, such as damaged cells, microorganisms, poisonous, and allergenic substances [147]. It serves as a crucial stage in the process of tissue regeneration, repair, and remodelling, as well as the restoration of tissue haemostasis in impaired areas [148]. Inflammatory mediators include the cytokines interleukin (IL)-1, IL-5, IL-6, IL-12, IL-1*β*, TNF-*α*, and interferon *γ* [149], and chemokines such as IL-8, monocyte chemoattractant protein 1, cyclooxygenase, vascular cell adhesion molecule 1, matrix metalloproteinase, free radicals, growth factors, and prostaglandins serve as regulatory mediators in the process of inflammation [150]. On stimulation, these mediators activate endothelial cells, causing increased vascular permeability and the deployment of neutrophils, eosinophils, monocytes, and mask cells to the injury site, which helps eliminate the harmful agents and facilitate the healing process [151]. However, inflammation is known to contribute to the development and progression of various diseases, including but not limited to CKD, cancer, diabetes mellitus, cardiovascular disease, NAFLD, obesity, asthma, rheumatoid arthritis, osteoporosis, autoimmune, and neurodegenerative disorders [152–154]. The consumption of natural antioxidants for maintaining human health has become popular, especially in developed nations [155]. In a study using female Wistar rats, lycopene was shown to alleviate palmitic acid-induced neuroinflammation by reducing oxidative stress and inhibiting the toll-like receptor 4 (TLR4) and nuclear factor kappa-B p65 (NF-kB p65) signalling pathways [156]. Lycopene supplementation mitigated metalaxyl-induced liver damage in male albino rats by restoring antioxidant status, improving liver function, and alleviating liver injury-associated complications [157]. In lycopene-treated endothelial cells, lycopene inhibited the activation of TNF-*α* but enhanced the expression of heme oxygenase-1 (HO-1) through the upregulation of nuclear factor erythroid 2-related factor 2 signalling pathways [158]. Another experimental study reported that in male albino rats, orally administered lycopene at 10 mg/kg body weight for 21 days effectively protected the colon epithelial mucosa against acetic acid-induced colitis and oxidative injury [159]. In C57BL/6 mice chronically exposed to cigarette smoke for 60 days, lycopene has shown to restore redox status and mitigate hepatic inflammation [160]. In addition, Li et al. [161] reported that lycopene mitigated the dysregulation of lipid metabolism and the inflammatory response induced by lipopolysaccharide in the rat testes. Thus, evidence is plentiful demonstrating the anti-inflammatory effects of lycopene both *in vitro* and *in vivo*.

### 5.8. Antidiabetic Effects

Diabetes mellitus (DM) causes hyperglycaemia and, if inadequately managed, can result in damage to the heart, eyes, and kidneys [162]. The global prevalence of diabetes is approximately 9.3%, which corresponds to about 463 million individuals. However, it is predicted to rise by 25% in 2030 and 51% in 2045 [163]. Diabetes mellitus is classified into three major types: type 1 (insulin-dependent), type 2 (noninsulin-dependent), and gestational diabetes mellitus [164]. Among these, type 2 diabetes mellitus predominates and accounts for about 90% in all cases worldwide [162].

Scientific evidence shows that lycopene can potentially be used to prevent and treat diabetes mellitus [24]. In streptozotocin-induced diabetes model, dietary fortification with lycopene mediated increased serum insulin concentrations, decreased urine and blood sugar concentrations, and reduced diabetes-induced pancreatic injury [165]. In diabetic Wistar rats, orally administered lycopene at 40 mg/kg body weight significantly decreased serum MDA, cortisol, and blood glucose concentration but increased SOD, CAT, and glutathione peroxidase (GSH-Px) activities at 10, 20, and 40 mg/kg body weight [166]. Furthermore, supplemental lycopene has shown to attenuate renal damage in diabetic rats [167]. In STZ-induced diabetic rats, at 4 mg/kg body weight, lycopene-ameliorated B-cell lymphoma-extra-large, and B-cell lymphoma 2 (Bcl-2) concentrations and reduce the expression of Bcl-2-associated X-protein (BAX) in the hippocampus [168]. Interestingly, orally administered lycopene has shown to increase SOD and GSH-Px activities and lower MDA concentrations in rat pancreatic tissue [169], but it mediated increased plasma insulin concentrations and reduced blood and liver lipid content, fasting blood glucose and glycosylated haemoglobin concentration, and homeostatic model assessment for insulin resistance in diabetic rats [169].

### 5.9. Anticancer Effects

Cancer is a major global health challenge and is the second primary reason for mortality in the United States [170]. The ingestion of tomatoes and tomato-based products has been associated with a reduced occurrence of different types of cancer [171]. *In vivo* and *in vitro* research has demonstrated that lycopene hinders the growth and multiplication of prostate cancer cells, inhibits the cell cycle, and induces apoptosis [172]. Dietary supplementation with lycopene mitigated the growth of breast cancer cells by suppressing the activity of the insulin-like growth factor 1 receptor (IGF-1R) signalling pathway [151]. While research shows that the consumption of a lycopene-rich diet could be beneficial in reducing the risk of pancreatic cancer [131]. In a rat model, the consumption of lycopene has shown to reduce the progression and proliferation of ovarian cancer [173], and in human studies, cisplatin-based chemotherapy in combination with lycopene supplementation enhanced cervical cancer treatment [174]. Furthermore, in animal models of hepatocellular carcinoma, administered lycopene suppressed the onset and development of cancer [175]. In human colorectal adenocarcinoma cell line, treatment with lycopene has shown to exhibit genotoxicity, antiproliferative, and apoptotic effects [176], a demonstration of its anticancer effects.

### 5.10. Gastroprotection

The incidence of peptic ulcer disease (PUD) has substantially increased, affecting approximately 5 to 10 percent of the general population [177]. The corrosive effects of acid and pepsin on the gastroduodenal mucosa cause peptic ulceration through exposure of the mucosa's lining to gastric acid and digestive enzyme actions [178]. Peptic ulcer disease is primarily caused by the extensive use of nonsteroidal anti-inflammatory drugs (NSAIDS) and *Helicobacter pylori* infection [179]. Other contributing factors include surgery, severe illness, burns, Zollinger–Ellison syndrome, excessive alcohol intake, smoking, and psychological and physical stress [180–182]. The excessive production of ROS is the major factor in stress-induced ulcers [183]. Thus, the utilisation of strong antioxidants may be beneficial in the management of ulcers [184]. In male Albino rats, lycopene administered at 200 mg/kg body weight for 10 days has shown to protect against ethanol-induced mucosal injury [185]. In their study, Chen et al. [186] found that supplemental lycopene at 10, 50, 100, and 150 mg/kg body weight reduced gastric juice secretion in adult male Kunming mice when compared to the gastric injury control group. However, at high doses (150 mg/kg body weight), lycopene exacerbated absolute ethanol-induced acute gastric mucosal injury. In addition to mediating for protection against alcohol-induced gastrointestinal tract mucosal injury, lycopene has shown to suppress gastric acid secretion and combat infection by *Helicobacter pylori* [130].

### 5.11. Neuroprotection

Neurodegenerative diseases (NDs) are characterised by gradual loss of neurons and are associated with the formation of protein aggregates [187]. These diseases are considered a major medical challenge as it affects millions of patients globally [188]. Alzheimer's, Parkinson's, Huntington's, prion and motor-neural diseases, amyotrophic lateral sclerosis, spinocerebellar ataxia, and spinal muscular atrophy are common NDs [187, 189, 190]. Despite age being the leading factor in the onset of all neurodegenerative disorders, recent discoveries indicate that the combination of a person's genetic makeup and environmental influences can contribute to an elevated risk of developing NDs [191]. Regardless of the various factors causing these NDs, a key feature common to all is the onset and development of neuronal cell death [192]. The progression of NDs is characterised by increased ROS production, which causes oxidative stress [193]. Administered lycopene has shown to attenuate memory loss due to age, cognitive impairments, neuronal damage, and synaptic dysfunctions in the brain [194]. In addition, lycopene was observed to mitigate age-related oxidative stress by suppressing lipid peroxidation and enhancing GSH, SOD, and CAT activities [194]. Dietary fortification with lycopene was demonstrated to decrease age-related neuroinflammation by attenuating microgliosis and combating inflammation [194]. Furthermore, lycopene mediated the reduction in the accumulation of amyloid beta 1–42 in the brains of aged CD-1 mice [194] and when used as a supplement, it upregulated the mitogen-activated protein kinase (MARK)/extracellular signal-regulated kinase (ERK) signalling pathway, inhibited oxidative stress and neuronal apoptosis, and protected against bisphenol-induced neurotoxicity in the hippocampi of adult male rats [195]. It has also shown to decrease palmitic acid-induced brain oxidative stress and neuroinflammation and to inhibit the toll-like receptor 4 (TLR4)/nuclear factor kappa-light chain enhancer of activated B cells p65 (NF-kB-p65) pathway in female rats [156]. In mice with Alzheimer's disease induced by *β* amyloid, lycopene reduced oxidative stress, decreased neuronal loss, improved synaptic plasticity, and inhibited neuroinflammation [196].

### 5.12. Cardioprotection

Globally, cardiovascular diseases (CVDs) stand at the forefront as the leading cause of human mortality [16]. Studies have shown that in 2019, CVDs caused 17.8 million fatalities, and this trend is projected to increase by 2030 to 23 million [197]. Several epidemiological studies have confirmed the significance of lycopene in preventing CVDs [198]. For instance, lycopene supplementation has shown to reduce C-reactive protein levels, interleukin-6, pulse wave velocity, blood pressure, and intercellular adhesion molecule 1 and enhance vascular health through flow-mediated dilation of the endothelium [199]. Lycopene supplementation at a dosage of 5 mg/kg body weight for 21 days has shown to confer protection against atrazine-induced cardiotoxicity in mice [200]. In Brown–Norway/Lewis rat model, lycopene treatment was demonstrated to have the potential to mitigate vascular arteriosclerosis in allograft transplantation by inhibiting Rho-associated kinases and by regulating the expression of nitric oxide/cyclic guanosine monophosphate signalling pathways [201], which indicates that lycopene has the potential to alleviate vascular arteriosclerosis. In another study, lycopene administered for 4 weeks at 10 mg/kg body weight reduced inflammation and apoptosis during postmyocardial infarction remodelling by suppressing the NF-KB signalling pathway in mice [202]. In addition, supplemental lycopene improves endothelial function in individuals suffering from CVDs [203].

### 5.13. Lung Protection

In male C57BL/6 mice, dietary lycopene supplementation at 25 or 50 mg/kg body weight mitigated cigarette smoke-induced pulmonary emphysema [204]. The literature shows that lycopene or matrine treatment alone offered minimal protection against lipopolysaccharide-induced acute lung injury in mice, but when coadministered, significant mitigatory effects were observed [205]. These results indicate that lycopene and matrine in combination may function as an alternative to glucocorticoid therapy in treating acute lung injury [205]. In a study conducted by Mustra Rakic et al. [206], supplemental lycopene at 90 mg/kg body weight for 22 weeks effectively suppressed tobacco carcinogen/cigarette smoke (NNK/CS)-induced emphysema, chronic bronchitis, and preneoplastic lesions. Furthermore, dietary lycopene significantly decreased NNK/CS-induced buildup of total cholesterol and upregulated mRNA expression of peroxisome proliferator-activated receptor alpha (PPAR*α*), ATP-binding cassette (ABC) transporters ABCA1 and ABCG1, and liver X receptor alpha (LXR*α*) in the lungs of the ferret model. These findings suggest that lycopene could act as a preventative agent against the adverse effects of tobacco smoke on lung health and lipid metabolism.

### 5.14. Sperm Quality Enhancement and Fertility Promotion

Infertility is a prevalent health problem that affects roughly 48 million couples and 186 million individuals globally [207]. ROS-induced oxidative stress is a primary contributor to various reproductive complications [208]. In varicocele-induced rats, supplemental lycopene has shown to protect sperm against DNA damage by mediating upregulation of antioxidant responses that quenched ROS, which manifested with improved sperm viability, Johnson's score, membrane integrity, and the expression of B-cell lymphoma 2-associated X-protein (BAX) [209]. Similarly, in men with oligozoospermia, supplemental lycopene for 12 weeks at 25 mg/kg body weight attenuated oxidative stress and improved sperm quality [52]. In their study, Yamamoto et al. [210] observed that the consumption of tomato juice with 30 mg of lycopene for a duration of 12 weeks increased plasma lycopene concentration and sperm motility and decreased the white blood cell count in the seminal plasma of the tomato juice group compared to the control group of infertile men. Dietary supplementation with lycopene at 20 mg per day for 3 months prior to the scheduled *in vitro* fertilization (IVF) treatment increased the arachidonic acid to docosahexaenoic acid ratio in the seminal fluid and resulted in 7 natural pregnancies in addition to 15 pregnancies following the IVF procedure [51]. In methotrexate-induced ovarian damage, pretreatment with lycopene at 5 mg/kg body weight for 5 days prevented infertility and has shown to mediate increased GSH activity as well as decreased MDA and myeloperoxidase concentrations [211]. These findings suggest that lycopene alleviates imbalances in polyunsaturated fatty acids and can serve as a preventive agent against infertility.

### 5.15. Protection of Skin Health

The skin, constituting approximately 15% of the total body weight [20], plays a vital role in preventing excessive water loss from the body and maintaining the body temperature within an optimal range [212]. It provides protection against toxic substances, free radicals, physical damage, and ultraviolet radiation [213]. The latter causes the development of skin conditions and diseases through sunburn, photoaging, and excessive ROS production within the skin, which damages DNA and causes skin cancer [213–215]. Lycopene is extensively used as an ingredient in cosmetic products due to its demonstrated ability to protect the skin from aging and photodamage [215]. Anbualakan et al. [216] showed that lycopene can prevent and/or treat sunburn and photoaging and that it could potentially be effective against UV-induced skin cancers. As a dietary supplement, lycopene has been demonstrated to improve skin appearance and pigmentation and mitigate erythema [217].

### 5.16. Protective Effect on Vision

Age-related ophthalmic conditions, inclusive of macular degeneration, glaucoma, cataracts, and diabetic retinopathy, are key contributors to gradual and permanent vision loss [218]. In diabetic patients, serum lycopene concentration has been observed to be lower than normal [114]. Importantly, due to its consistent lower levels in diabetics, it has been suggested that serum lycopene concentration might serve as a diagnostic tool for diabetic retinopathy [114]. Using ARPE-19 cells derived from human retinal pigment epithelium, Gong et al. [219] demonstrated that lycopene suppressed growth of human RPE cells against oxidative stress-induced cell loss findings which suggests that it (lycopene) may protect against RPE proliferative disease and old-age related macular degeneration. Oxidative stress and inflammation have been shown to be associated with the pathogenesis of eye-related conditions [220]. As a dietary supplement, lycopene has been proven to mitigate the risk of developing eye diseases associated with old age [221]. This could be due to its demonstrated ability to prevent cataract formation both *in vitro* and *in vivo* [131].

## 6. Lycopene: Protective Effects against Toxins

Toxins are natural and harmful chemical substances that adversely impact health [222]. They cause specific organ toxicity, for example, skin, eye, kidney, liver, blood, cardiovascular, respiratory, reproductive, endocrine, immune, and nervous system damage [222, 223]. Through their actions, toxins disrupt homeostasis, alter gene expression, and cancer-related metabolic signalling pathways [224]. Research has demonstrated that lycopene as a dietary supplement effectively mitigates the deleterious effects of myco-, bacterial, and chemical toxins [225] [125, 226, 227], fungicides [228], pesticides [229], herbicides [230], and fluoride [231]. It is hypothesised that lycopene mediates protection against toxins through its potent antioxidant, chelating, and antiapoptotic properties [224].

## 7. Lycopene: Safety and Potential Toxicity

There is no specified daily prescription for dietary lycopene intake, but epidemiological studies have recommended an intake of 2 to 20 mg daily of lycopene [93]. It has shown that consumption of up to 100 mg of lycopene daily does not elicit adverse outcomes [5]. In a toxicological study conducted on rats, feeding a diet fortified with lycopene at 1% (w/w) did not elicit any side effects [232]. Similarly, using lycopene at 200 mg/kg body weight per day as a dietary supplement has also been shown not to negatively impact animals [233]. Generally, it is asserted that lycopene can be used as a safe dietary supplement during pregnancy and lactation [234]. Although in pregnant women, high dietary intake of lycopene has shown to mitigate the risk of developing preeclampsia [235]. Imran et al. [7] reported that excessive chronic consumption of tomato juice, a rich source of lycopene, caused lycopenemia. Findings from both animal and human studies suggest that although lycopene could generally be used as a safe dietary supplement, some caution must be exercised against excessive intake.

## 8. Conclusion

The extensive studies carried out on lycopene highlight its exceptional potential to promote overall health and well-being. Its varied spectrum of benefits places it as a potent natural compound which can contribute to the promotion of health either as a prophylactic or ore therapeutic agent against metabolic diseases. In order to fully exploit its potential and increase its utility in health delivery, it is crucial to undertake additional research to comprehensively elucidate the health beneficial mechanisms underlying lycopene's medicinal properties. Furthermore, in order to enjoy optimal utility from the use of lycopene, there is a need to evaluate and recommend effective dosages for efficacy and prevention of possible side effects of abnormally high doses.

## Figures and Tables

**Table 1:** Lycopene concentration in fresh fruits and processed food products.

Fruit/processed food product	Lycopene content (mg/100 g)
Apricot and fresh tomatoes	0.11–5.3
Carrot	0.65–0.78
Cooked tomatoes	3.70
Fresh tomatoes	0.72–4.2
Ketchup	9.90-13.44
Papaya	0.11–5.3
Pink grapefruit	0.35–3.36
Pink guava	5.23–5.5
Pumpkin	0.38–0.46
Rosehip	0.68–0.71
Sweet potato	0.02–0.11
Tomato paste	5.40–150
Tomato sauce	6.20
Watermelon	2.30–7.20

Source: [9, 10].

**Table 2:** Physical properties of lycopene.

Property	Value/normal range
Boiling point	660.9°C at 760 mmHg
Crystal form	Long red needles separate from a mixture of carbon disulfide and ethanol
Density	0.889 gm/cm^3^
Flash point	350.7°C
Main hazards	Combustible
Melting point	172–175°C
Molecular weight	536.85 Da
Powder form	Dark reddish-brown
Refractive index	1.531
Solubility	Soluble in chloroform, hexane, benzene, carbon disulfide, acetone, petroleum, tetrahydrofuran, carbon disulfide, ether, and oil; insoluble in water, ethanol, and methanol
Stability	Sensitive to light, oxygen, high temperature, acids, catalyst, and metal ions
Vapour pressure	1.33·10−16 mmHg (25°C)

Source: [8, 29].

**Table 3:** Lycopene concentration in some human tissues.

Tissue	Lycopene (nmol/g wet weight)
Adipose	0.2–1.3
Adrenal	1.9–21.6
Brainstem	Non detectable
Breast	0.8
Colon	0.3
Liver	1.3–5.7
Lung	0.2–0.6
Ovary	0.3
Prostate	0.8
Skin	0.4
Stomach	0.2
Testis	4.4–21.4

Source: [36, 37].

**Table 4:** Meta-analyses and clinical trials of lycopene in human studies.

Biological effects	Mechanisms of action	References
Anticancer	Suppressed cell proliferation, induced cell cycle arrest, and increased apoptosis in breast cancer cell lines	[40]
	Decreased insulin-like growth factor-1 (IGF-1) and increased apoptosis in prostate cancer cell	[41]

Cardioprotection	Enhanced endothelial function and decreased triglyceride levels in patients with ischemic heart failure	[42]
	Increased flow-mediated dilation and total oxidative status decreased	[43]
	Increased HDL, paraoxonase-1 (PON-1), lecithin cholesterol acyltransferase (LCAT), decreased serum amyloid A (SAA), and cholesteryl ester transfer protein (CETP) activities	[44]

Antidiabetic	Reduced levels of fasting blood glucose in patients with type 2 diabetes mellitus	[45]
	Decreased glycated haemoglobin (HbA1c) levels and fasting blood glucose concentration	[46]

Anti-inflammatory	Inhibited NF-kB and c-Jun N-terminal kinase (JNK) activation. Suppressed the expression of COX-2, iNOS, TNF-*α*, IL-1*β*, and IL-6	[47]

Antioxidant	Increased bone mineral density	[48]
	Increased SOD, GSH-px, and decreased MDA	[49]

Sperm quality enhancement and fertility promotion	Decreased lipid peroxidation and fragmentation of sperm DNA	[50]
	Increased arachidonic acid to docosahexaenoic acid ratio	[51]
	Reduced oxidative stress and enhanced sperm quality	[52]

Hepatoprotection	Protection against steatosis and liver damage	[53]
	Regulated oxidative stress and liver enzyme levels in individuals with metabolic syndrome	[54]

Antiobesity	Decreased body weight, BMI, waist circumference, total cholesterol, and increased HDL levels	[55]

Renoprotection	Elevated levels of serum lycopene reduce the risk of mortality in individuals with CKD	[56]
	Increased consumption of lycopene decreased the occurrence of CKD in women	[57]

Lung protection	Decreased airway neutrophil influx and decreased activity of neutrophil elastase in sputum	[58]
	Increased SOD and CAT and decreased MDA, TNF-*α*, IL-1*β*, and IL-6 levels in chronic obstructive pulmonary disease (COPD)	[59]

Neuroprotection	Elevated serum levels of lycopene are associated with a decreased risk of Alzheimer's disease (AD) mortality in adults	[60]
	Enhanced cognitive function in middle age	[61]

Gastroprotection	Decreased bleeding index and reduction in the percentage of gingivitis	[62]
	Increased consumption of lycopene improved bowel function and helped prevent chronic constipation	[63]

Osteoprotection	Stimulated WNT/*β*-catenin and ERK1/2 pathways, increased the expression of RUNX2, alkaline phosphatase, and COL1A, and decreased RANKL in Saos-2 cells	[64]
	Increased total antioxidant capacity, decreased lipid peroxidation, protein oxidation, and N-telopeptide of type 1 collagen	[65]

## Data Availability

The data that support this systematic review come from studies and datasets that were previously reported and cited in this article.
